# The Role of Dietary Antioxidants in the Pathogenesis of Neurodegenerative Diseases and Their Impact on Cerebral Oxidoreductive Balance

**DOI:** 10.3390/nu12020435

**Published:** 2020-02-08

**Authors:** Anna Winiarska-Mieczan, Ewa Baranowska-Wójcik, Małgorzata Kwiecień, Eugeniusz R. Grela, Dominik Szwajgier, Katarzyna Kwiatkowska, Bożena Kiczorowska

**Affiliations:** 1 Department of Bromatology and Food Physiology, University of Life Sciences in Lublin, 20-950 Lublin, Poland; malgorzata.kwiecien@up.lublin.pl (M.K.); eugeniusz.grela@up.lublin.pl (E.R.G.); kwiatkowska.katarzyna@up.lublin.pl (K.K.); bozena.kiczorowska@up.lublin.pl (B.K.); 2 Department of Biotechnology, Microbiology and Human Nutrition, University of Life Sciences in Lublin, 20-950 Lublin, Poland; ewa.baranowska@up.lublin.pl (E.B.-W.); dominik.szwajgier@up.lublin.pl (D.S.)

**Keywords:** neurodegenerative diseases, exogenous antioxidants, diet, prevention

## Abstract

Neurodegenerative diseases are progressive diseases of the nervous system that lead to neuron loss or functional disorders. Neurodegenerative diseases require long-term, sometimes life-long pharmacological treatment, which increases the risk of adverse effects and a negative impact of pharmaceuticals on the patients’ general condition. One of the main problems related to the treatment of this type of condition is the limited ability to deliver drugs to the brain due to their poor solubility, low bioavailability, and the effects of the blood-brain barrier. Given the above, one of the main objectives of contemporary scientific research focuses on the prevention of neurodegenerative diseases. As disorders related to the competence of the antioxidative system are a marker in all diseases of this type, the primary prophylactics should entail the use of exogenous antioxidants, particularly ones that can be used over extended periods, regardless of the patient’s age, and that are easily available, e.g., as part of a diet or as diet supplements. The paper analyzes the significance of the oxidoreductive balance in the pathogenesis of neurodegenerative diseases. Based on information published globally in the last 10 years, an analysis is also provided with regard to the impact of exogenous antioxidants on brain functions with respect to the prevention of this type of diseases.

## 1. Introduction

Neurodegenerative diseases are progressive diseases of the nervous system that result in neuron loss or functional disorders. The common denominator in most such diseases pertains to changes in the function of glial cells in the brain (astrocytes, oligodendrocytes, and microglia), which regulate inflammation and cell metabolism disorders [1]. The increasing incidence of neurodegenerative diseases can be observed worldwide. This is due to both the general ageing of societies and unhealthy lifestyle [2]. Studies reveal that the lowest numbers of neurodegenerative patients are observed in Asia, which is due to the prevalence of diets based on fresh plant products containing a wide range of agents with string antioxidative effects: fruit, vegetables, and spices, as well as the wide-spread consumption of tea [3]. Said substances modify the multistage signal transduction pathways [3].

Neurodegenerative diseases require long-term, sometimes life-long pharmacological treatment, which increases the risk of adverse effects and a negative impact of pharmaceuticals on the patients’ general condition. Moreover, one of the main problems related to the treatment of this type of condition is the limited ability to deliver drugs to the brain due to their poor solubility, low bioavailability, and the effects of the blood-brain barrier [4]. This may be the reason why no effective treatment has yet been developed that would allow reversal of dementia (in the sense of restoring correct cognitive functions) or at even inhibition of further degenerative progression, i.e., clinical stabilization of the patient. The currently available pharmacotherapy in cases of dementia facilitates only a temporary improvement of the patients’ cognitive functions by reducing the severity of behavioral disorders and psychiatric symptoms related to dementia and general improvement of their everyday wellbeing [5]. Given the above, one of the main objectives of contemporary scientific research focuses on the prevention of neurodegenerative diseases. That goal, however, is not easily attained, as there are two necessary conditions that have to be met for a treatment to be successful: (1) increased-risk patients need to be diagnosed before the symptoms of the disease become apparent and (2) such persons need to be provided with adequate prophylactics aimed at reducing the risk or slowing down the onset of the disease [3]. As disorders related to the competence of the antioxidative system are a marker in all diseases of this type [3], the primary prophylactics should entail the use of exogenous antioxidants, particularly ones that can be used over extended periods, regardless of the patient’s age, and ones that are easily available, e.g., as part of a diet or as diet supplements. The paper analyzes the significance of the oxidoreductive balance in the pathogenesis of neurodegenerative diseases. Based on information published globally in the last 10 years, an analysis is also provided with regard to the impact of exogenous antioxidants on brain functions with respect to the prevention of this type of disease. 

## 2. The pathogenesis of Neurodegenerative Diseases

Neurodegenerative diseases are caused by neuron loss or disorders in neuron functions, with the common denominator being the changes in the function of glial cells in the brain (astrocytes, oligodendrocytes, and microglia) and cell metabolism disorders. The functions of astrocytes and microglia are very closely related and directly influence the activity and survival of cerebral neurons. Astrocytes constitute the primary system of neuron support as the only cells capable of storing energetic reserves in the form of the glycogen, they provide neurons with energetic substrates in the form of lactate, protect them by producing antioxidants, and releasing growth factors [6]. Astrocytes are activated in response to oxidative stress or damage and adjust their functions accordingly by producing agents facilitating survival and regeneration [6]. Hence, a long-term disorder in astrocyte function may negatively influence the functioning of neurons. In turn, microglia are mainly responsible for cerebral inflammation as when activated, they destroy pathogens, remove the remains of dead cells and neutralize toxic protein aggregates, as well as release trophic agents to protect neurons [7]. The mutual interaction between astrocytes and microglia is the basis of their correct functioning in the nervous system. Oligodendrocytes are found both in the white and grey brain matter as well as in the spinal medulla. The cells are present in the vicinity of neurons, other types of neuroglia and blood vessels [8]. Due to their role, they arrange themselves next to nerve fibers as the cells producing their surrounding myelin. In the brain, oligodendrocytes also provide the scaffold for neurons and control water and electrolyte homeostasis [9]. After passing through the blood-brain barrier, iron is bound by transferrin produced by oligodendrocytes and epithelial cells of the choroid plexus. In that form, iron is circulated in the interstitial fluid and supplied to nervous system cells, whereas free Fe^2+^ ions can participate in the formation of free radicals through Fenton and Haber-Weiss reactions, including the particularly toxic hydroxyl radical [10].

The pathogenesis of neurodegenerative diseases involves numerous factors; however, the key roles are played by inflammatory factors and oxidative imbalance. The same applies to, e.g., Alzheimer’s disease, Parkinson’s disease, Huntington’s disease, amyotrophic lateral sclerosis, and multiple sclerosis [3]. 

Alzheimer’s disease (AD) stems from multifactor neurodegenerative disorders; it is a primary degenerative disease of the brain caused by the deposition of proteins with pathological structures (β-amyloid, Tau protein, and α-synuclein), which causes neuron death and loss of inter-neuron connections [11]. Currently, two main causes of the disease have been suggested: β-amyloid cascade and degeneration of the cytoskeleton [12]. Characteristic histopathological symptoms include accumulation of amyloid plaques in the cerebral cortex and pathological Tau proteins in neurons and neuroglia [11]. The gradual degeneration and atrophy of neurons is accompanied by the formation of non-physiological protein species capable of aggregation and resistant to the effects of proteolytic enzymes, as well as damage to signal transduction pathways, resulting in a decreased level of relay substances, of which the reduction in the acetylcholine content due to the effects of acetylcholinesterase (AChE) and its decomposition into choline and acetic acid residue is the most important for the memory system [13]. Excessive induction of astrocytes and microglia takes place, accompanied by phagocytosis and excretion of multiple inflammatory factors such as: Cytokines, reactive oxygen radicals, and nitrogen oxide (NO). Hyperactivation of microglia and astrocytes induces neuron apoptosis and damages the blood-brain barrier which is crucial to the integrity and correct functioning of the nervous system [14]. The processes stimulate astrocytes to produce proinflammatory proteins, reactive oxygen species (ROS), and NO. This facilitates the formation of insoluble β-amyloid (Aβ), which shows neurotoxic properties [12]. Alzheimer’s disease is accompanied by the development of cerebral inflammation with the symptoms including the following markers: (1) increased activity of α-1-antichymotrypsin and α-1-antitrypsin, which indicate an active inflammation; (2) elevated concentration of proinflammatory cytokines IL-1b and IL-6 released by microglia; and (3) increased lipid peroxidation induced by free radicals inducing oxidative stress [12,14,15]. The concentration of carbonyl groups (which are the main markers of protein oxidation) in the substantia nigra of AD patients was twice as high as that observed in healthy subjects [16]. Three times more damage has been observed in the mitochondria of AD patients as compared to healthy persons; they also showed reduced activity of cytochrome oxidase in the frontal and temporal cortex, which leads to the accumulation of the products of incomplete oxygen reduction, in particular the hydroxyl radical [7].

Mitochondrial damage is considered to be the key factor determining the pathomechanism of Parkinson’s Disease (PD) [17]. The disease is caused by gradual atrophy of dopaminergic neurons in the substantia nigra, which leads to dopamine deficiency in the striatum [18]. Dopamine is an important neurotransmitter synthesized and released by dopaminergic neurons of the central nervous system. For that reason, PD treatment should focus on increasing production and/or release of dopamine, inhibiting dopamine metabolism, and stimulating dopaminergic receptors [19]. The pathomorphological characteristics of PD reveals the intraneuronal presence of Lewy’s bodies, which leads to neuron death caused by the mechanism of apoptosis [18]. A number of factors have to be taken into account in the context of PD, including both genetic (the first identified mutation was of the α-synuclein gene PARK1 in chromosome 4) and environmental, in particular the effects of free radicals and oxidative stress [17]. It has been demonstrated that the concentration of lipid peroxidation products was eight times higher in patients suffering from Parkinson’s disease as compared to healthy subjects [7]. The contribution of oxidative stress to PD is also evidenced by the increased levels of nucleic acids and protein oxidation products, as well as decreased concentrations of reduced glutathione and antioxidative enzymes when compared to healthy persons [18]. The production of excessive amounts of ROS is facilitated by high iron concentrations [17].

Huntington’s disease (HD) is caused by a mutation entailing the presence of an increased number of copies of three CAG nucleotides in the IT15 gene. As a consequence, the abnormal mHtt protein is formed (a mutated version of the Huntingtin protein), which leads to irreversible neuronal damage, mainly in the basal ganglia. Huntington’s disease is inherited through an autosomal dominant mutation and is characterized by a high degree of penetrance [20]. Experimental data have been published that indicate the key role of mitochondrial dysfunction in the pathogenesis of HD. As observed in those studies, a key factor influencing the toxic effects of mHtt is the direct or indirect interaction between this protein and mitochondria, most likely triggering changes in mitochondrial membranes, which leads to increased production of ROS and dysfunctions of the respiratory chain [21]. One of the primary tools in the exchange of metabolites between mitochondria and cytoplasm is the VDAC (voltage dependent anion selective channel). It has been demonstrated that VDAC intermediates in the determination of the oxidative-reductive state of cytosol, which in turn serves and an important factor determining the synthesis level of proteins eliminating the superoxide anion radical (superoxide dismutase Mn-SOD and Cu, Zn-SOD) and protein included in the import complexes of the external mitochondrial membrane [22]. An increasing number of studies currently suggest that dyshomeostasis of transition metals may constitute a part of HD pathogenesis. In particular, iron (Fe) and copper (Cu) play the roles of the pathology’s mediators. A significantly increased concentration of Fe and Cu was observed in brain tissue collected post-mortem from the brains of HD patients, as well as in the cerebral tissues of R6/2 mice and in the Drosophila HD model [21]. Increased accumulations of Fe were also observed in the ganglia of the base of the brain and cortex of HD patients, as well as elevated malondialdehyde (MDA) levels in the blood and 8-hydroxyguanine in the brain [7]. 

Multiple sclerosis (SM, Sclerosis multiplex) is a chronic, inflammatory degenerative disease of the central nervous system. It is characterized by multifocal and time-scattered emergence of inflammatory demyelinative lesions causing damage to and loss of axons [23]. The pathogenesis of the disease is complex and remains not fully characterized, with a number of key factors mentioned in this context: damage to the blood-brain barrier, emergence of multifocal perivascular cellular infiltrations, and damage to the myelin and loss of axons and oligodendrocytes, as well as secondary astroglial hypertrophy [23,24]. Leukocyte rolling from blood to the central nervous system results in the activation of microglia that release proinflammatory cytokines, which have phagocytic properties and promote oxidative stress, which leads to extensive damage, primarily to deep white matter in the region of optic nerves, corpus collosum, and periventricular matter, as well as in subtentorial regions and in the vicinity of the spinal cord, particularly in its cervical section. The inflammatory process is the driving force of the demyelination [24].

Amyotrophic lateral sclerosis (ALS) belongs to the group of motor neuron diseases. It is a primarily degenerative disease of the nervous system with a progressive course and thus far undiscovered etiology. It is caused by neuron atrophy. It is currently assumed that the primary role in ALS etiopathogenesis is played by genetic factors, primarily mutations of genes conditioning SOD-1 (superoxide dismutase) synthesis [25]. SOD is an enzyme found in cytosol and mitochondria, it catalyzes the reaction of superoxide anion radical dismutase, which leads to the formation of hydrogen peroxide and molecular oxygen through reduction and oxidation of metals contained in the centers of active SODs—zinc and copper [10]. Patients show elevated levels of products of lipid, protein and DNA oxidation as well as H_2_O_2_ and the hydroxyl radical [25,26]. Clinical symptoms of ALS include simultaneous emergence of signs of upper motor neuron and lower motor neuron damage [27]. 

The pathogenesis of neurodegenerative diseases involves numerous factors; however, the competence of the antioxidative system are a marker in all diseases of this type. Astrocytes are activated in response to oxidative stress and adjust their functions accordingly by producing agents facilitating survival and regeneration.

## 3. Oxidative Stress as the Primary Cause of Brain Damage

**Figure 1:**
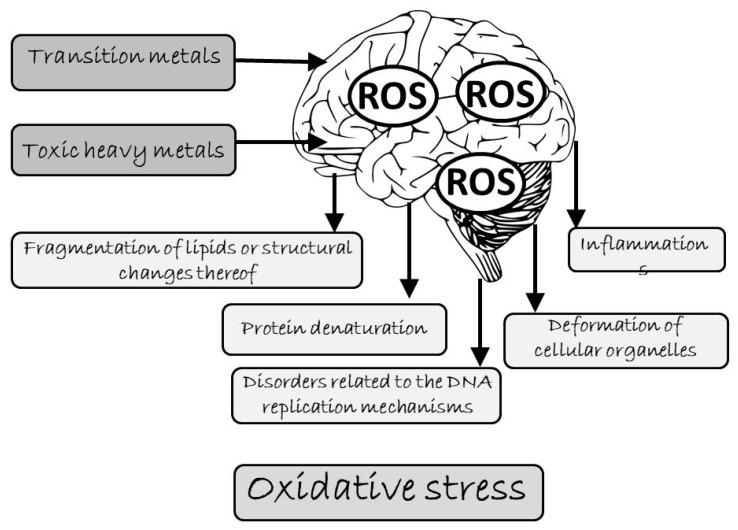
Prooxidative effects of metals on the brain (ROS - reactive oxygen species).

Oxidative stress is described as a condition in which the cellular antioxidative defenses prove insufficient due to excessive release of oxidants [10]. It can occur locally. The fact that antioxidative defenses have been overcome in a given organ or tissue does not influence the antioxidative activity in the rest of the organism. The mechanisms of antioxidative defenses are specific to particular ROS. The primary consequences of oxidative stress include fragmentation of lipids or structural changes thereof, protein denaturation, disorders related to the DNA replication mechanisms, and deformation of cellular organelles, and consequently, entire cells (Figure 1). ROS-induced oxidative stress leads not only to inflammation but also triggers the NF- κB (nuclear factor kappa-light-chain-enhancer of activated B cells) protein-dependent transcription of genes for various proinflammatory factors [28]. 

The activity of ROS leads to various types of intracellular damage, including enzyme activation, DNA damage, structural changes in protein and carbohydrate molecules. Moreover, ROS react with many unsaturated fatty acids on cell membranes, which initiates the process of lipid peroxidation resulting in modification of proteins and changes to the membrane gradient, which in turn leads to loss of integrity and irreversible damage [10,29]. The presence of an unpaired electron means that such molecules are characterized by high reactivity as they tend to pair-up their electrons by way of either donating or accepting one. Increased content of ROS inside cells may also result from weakening of their antioxidative mechanisms, mainly due to decreased intracellular concentrations of reduced glutathione (GSH), total pool of SH-groups with bound proteins, and changes to the activity of antioxidative enzymes [10]. Oxidatively modified compounds interfere with the neuron homeostasis, which may lead to their death due to apoptosis or necrosis [30]. A particular problem relates to the fact that the nervous tissue of the central nervous system possesses poor regenerative capacity, and despite continuous exposure to oxidative stress, has not developed effective mechanisms of minimizing its effects as no elevated levels of endogenous antioxidants capable of compensating for the increased ROS levels have been observed in the nervous system [31].

The brain is an organ particularly susceptible to oxidative modifications. The same is caused by its high demand for oxygen (approximately 20% of the organism’s total oxygen intake) and high content of lipids and transition metals (e.g., copper and iron), as well as relatively low levels of antioxidative enzymes [32]. Approximately 5% of the oxygen used in the mitochondria, peroxisomes, and microsomes of the respiratory chain is converted into ROS [7]. Due to their shape, neurons are characterized by a very disadvantageous ratio of surface area to volume, while cell membranes are the most susceptible to the effects of ROS as they can suffer changes to, e.g., their fluidity as a result of lipid peroxidation and oxidation of the thiol groups in membrane proteins [7]. Oxygen related processes taking place within neurons are highly intensified due to the high content of unsaturated fatty acids and relatively low content of exogenous antioxidants, hence, even short-lasting hypoxia can cause an increase in ROS levels and damage to lipids, proteins, and DNA [15]. Particularly important changes in the activity of antioxidative enzymes are observed in cerebral mitochondria, which are the main source of superoxide anion radical and hydrogen peroxide [33]. The antioxidative ad detoxifying system of neurons is inefficient; therefore, most of their defensive functions are performed by astrocytes which regulate he oxidoreductive balance by storing and releasing endogenous antioxidants: glutathione and ascorbic acid [6]. Due to the rapid pace of metabolic processes, the cerebral cortex constitutes the main site of free radicals’ production. Therefore, it is in that region that we often observe the initial increase in the activity of SOD, catalase (CAT) glutathione peroxidase (GPX), and glutathione reductase, which is considered to be the adaptative response to oxidative stress induced by external factors [10,34]. As a result of long-term oxidative stress, the cellular activity of those enzymes is decreased, which has associated with oxidative inactivation of the active enzyme center or modifications to the enzymatic protein molecule, reduction of the enzyme synthesis speed, accumulation of peroxides, or all said factors jointly [10,35]. Catalase catalyzes the reaction of hydrogen peroxide disproportioning as well as the oxidation of substances such as methanol, ethanol, formate, nitrates, and quinones [36]. In mammal tissues, CAT is located mainly in the liver, erythrocytes, kidneys, and central nervous system, where the enzyme is present in the highest and comparable amounts in the cerebellum and spinal cord [37,38]. 

In human brain there are a number of free oxygen radicals present, particularly, superoxide amino radical O_2_^-^, hydrogen peroxide H_2_O_2_, hydroxyl radical •OH, and nitrogen oxide NO. Superoxide amino radical is not one of the most reactive ROS but it nonetheless has the ability to oxidize transition metal ions, which may result in the inactivation of antioxidative enzymes whose metals are cofactors [10]. It also has the ability to oxidize cysteine, which leads to modifications of protein structure and may deprive certain enzymes of their bioactivity [39]. A significantly stronger antioxidant is the protonated form of the superoxide anion radical—the hydroxide radical HO_2_^-^ which penetrates cell membranes and is the main initiator of lipid peroxidation [40]. Large amounts of hydrogen peroxide are produced in phagocytizing microglial cells during the so-called “oxygen explosion”. It is a very weakly reactive ROS, it does not oxidize membrane lipids or DNA directly but may oxidize thiol, phenol, thioester, and indole groups in various compounds [32]. The most aggressive ROS include the hydroxyl radical which may act both as a redactor and an oxidizer [41]. Thanks to its high reactivity and low substrate specificity, it can attack all molecules it comes in tough with inside a cell; therefore, it damages protein by oxidizing amino acid residues and sulfhydryl groups. It also modifies nitrogen bases in DNA. Fatty acids are particularly vulnerable to its activity, which is especially dangerous in cerebral tissue, 60% of which is composed of lipids [42]. As the main component of cell membranes, lipids play a vital role in maintaining the structural integrity of cells. Excessive lipid oxidation changes the physical properties of cell membranes and may lead to covalent modification of proteins and nucleic acids. In the brain, the hydroxyl radical inhibits the activity of monoamine oxidases, enzymes responsible for the catabolism of neurotransmitters such as dopamine, noradrenaline, and serotonin [7]. Moreover, it causes neuron loss in cerebral ischemia in the course of neurodegenerative diseases. The reaction of the hydroxyl radical with dopamine produces 6-hydroxydopamine, which is considered to be the main factor responsible for the pathogenesis of Parkinson’s disease [7]. Nitrogen oxide NO serves the role of a neurotransmitter and neuromodulator in the brain, but because of its free radical character, it can also have toxic effects, as evidenced by the observed elevated levels of NO in the brains of multiple sclerosis patients [43]. Excessive NO production can lead to neuron degeneration since it acts as an inhibitor of cytochrome oxidase, which serves as the last enzyme in the respiratory chain. Nitrogen oxide also participates in the oxidation of active cysteine residues in neuronal protein kinase C, while the number of the disulphide bridges formed depends on the intensity of oxidative stress [43]. As a result of the reaction between nitrogen oxide and superoxide anion radical, highly reactive peroxynitrite is formed, which, e.g., damages the phospholipids in synaptic membranes, activates apoptosis in neurons, and inhibits apoptosis in astrocytes [7]. 

The brain provides conditions which are conducive to Fenton’s reaction leading to the formation of the hydroxyl radical [10]. This is because cerebral tissue (particularly in basal ganglia and the extrapyramidal system) has a tendency to store excessive transitional metals (mainly iron and copper) that catalyze this reaction. The accumulation of iron and copper ions in the brain also facilitates autooxidation of certain neurotransmitters, e.g., dopamine, serotonin, or noradrenaline (the ions are reaction catalysts), which results in their functional impairment. 

ROS exert a negative impact on the brain through lipid peroxidation and damage to proteins and nucleic acids. Up to 60% of the human brain is composed of lipids, most of which are membrane phospholipids with unsaturated fatty acid residues [42]. Arachidonic acid (11:6n, DHA) and docosahexaenoic acid (22:6n; DHA) are particularly susceptible to peroxidation, and the two compounds constitute the main pool of fatty acids in the brain. Peroxidation of DHA produces neuroprostanes, which are the biochemical markers of endogenous free radical peroxidation of lipids [44]. The high iron content in certain cerebral structures is an additional factor stimulating peroxidation, but we still do not know the exact mechanisms of interaction between key cells in the central nervous systems: neurons, oligodendrocytes, astrocytes, and microglia in the regulation of iron metabolism [15]. Studies conducted by Yen and Hsieh [45] demonstrated the inhibitive influence of catecholamines, particularly dopamine, on the process of linoleic acid peroxidation. Catecholamine neurotransmitters are released in synapses; in the event of neuron death their production is dramatically decreased, which leads to reduced efficiency of nerve impulse transmission [46]. Proteins that are oxidatively damaged typically lose their bioactivity due to the aggregation of incorrectly folded proteins (e.g., Cu,Zn-SOD), which may be deposited outside neurons in the form of senile plaques mainly in the areas of the entorhinal cortex, hippocampus, prosencephalon, and amygdala, i.e., regions of the brain that are responsible for memory, learning, and emotions [7]. As a result of neurodegeneration, the mass of the temporal and frontal lobes is reduced, as is the case in Alzheimer’s disease [47]. Cholinergic and glutamatergic neurons are particularly susceptible to neurodegeneration, although death of other nerve cells has also been reported. In a correctly functioning cell, the process of folding results in a stable spatial structure of polypeptide, which allows it to reach full biological activity [7]. Elevated levels of oxidative stress due to the fact that metal ions are bound by an incorrectly folded SOD are observed in cased of amyotrophic lateral sclerosis where most likely, Cu ions are bound in the position of Zn [31].

Some of the most dangerous types of cell damage are caused by reactions between ROS and nuclear DNA. Such interactions may lead to single or double ruptures in the DNA, the latter being particularly toxic for a cell and capable of directly triggering cell death; they can also lead to the emergence of networking bonds or modifications to nitrogen bases [48,49]. Furthermore, attention has been drawn to damaged mitochondrial DNA (mtDNA), which may be a significant element in the etiology of many diseases and senile conditions [50]. Hydrogen peroxide and superoxide anion radical initiate DNA damage by interacting with ions of organic compounds containing metals (iron, copper) via Fenton’s reaction, which leads to the formation of •OH [10]. Hydroxyl radical, one of the most reactive oxidizers, is probably one of the most important causes of DNA damage. To date, the best researched oxidative DNA modification with mutagenic properties is 8-Oxo-2’deoxyguanosine (8-oxo-dG or 8-OH-dG) [51]. In the human brain, a clear increase in 8-oxo-dG has been observed in the mtDNA of persons aged 42–97 years, with particularly high accumulations observed in persons over 70 years old [52]. In the cited study, the ratio of 8-oxo-dG in mtDNA to nuclear DNA was 10 in subjects under 70 years of age and increased to 15 in persons over 70. Mitochondria are the main source of free radicals and the pace of DNA oxidation in these structures can be significantly higher when compared to the DNA in cell nuclei. Mitochondrial DNA is particularly susceptible to the effects of ROS mainly because mitochondria are responsible for the consumption of approximately 90% of all oxygen processed by the organism, with 1–2% of the metabolized oxygen being converted into free radical forms. Furthermore, mtDNA molecules are located in the vicinity of the inner mitochondrial membrane whose electron transport system is conducive to ROS production; mtDNA is not bound to histone proteins, and mitochondrial genes are less protected by the DNA repair system when compared to nuclear DNA [46,50]. Other common changes to mtDNA include deletions; in human fibroblasts exposed to ROS one of the most common deletions, mtDNA^4977^, is accumulated [53].

## 4. Ion Metals Stimulating Cellular Oxidation 

### 4.1. Transition Metals

Due to the absence of unpaired electrons in the outermost orbital layer, transition metals show a dynamic capacity for easy electron donation or acceptance, as described by the formulas Cu^+^ 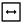 Cu^2+^ + e– and Fe^2+^ 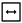 Fe^3+^ + e–. Their chemical character allows some of the compounds to take part in a range of physiological redox reactions [54]. They are involved in the formation of HO• from H_2_O_2_ in the course of Fenton’s reaction (they can catalyze Haer-Weiss reaction) and initiate non-specific lipid peroxidation [10]. The mechanisms of forming free radicals with the participation of transition metal ions are illustrated by Fenton’s and Haber-Weiss reactions [55]. In Fenton’s reaction, hydrogen peroxide disintegrates in the presence of transition metal (Me) ions (Fe^2+^, Cu^2+^) creating a hydroxyl radical:

Me^2+^ + H_2_O_2_ → Me^3+^ + OH• + OH^-^

The oxidized metal ion is reduced, and the superoxide anion radical is oxidized to molecular oxygen: 

Me^3+^ + O_2_•- → Me^2+^ + O_2_

The produced metal ion Me^2+^ can once again react with H_2_O_2_ and initiate the formation of progressively greater amounts of hydroxide radicals; Fenton’s reaction combined with the reaction of Me^3+^ ions’ reduction is described as a Haber-Weiss reaction:

O_2_•^-^ + H_2_O_2_ → O_2_ + OH• + OH^-^

It is noteworthy that the reaction of copper ions is biologically far less important when compared to iron reduction (except in cases of copper metabolism disorders), as the physiological cellular concentration of free copper is very low (at most, one atom per cell) [56]. Additionally, the ions of other metals such as chromium, cobalt, nickel, or manganese can behave in a similar way, i.e., produce a hydroxyl radical via Fenton’s reaction. However, the same only becomes important in cases of metal poisoning but have no physiological significance that would be even remotely comparable to the role of iron. 

Disorders of iron homeostasis in the central nervous system lead to the excessive accumulation of the metal in various cerebral structures. Oxidative stress, which intensifies in the presence of free iron ions, plays a key role in the pathogenesis of neurodegenerative diseases [54]. It has been demonstrated, however, that the level of iron in the central nervous system is not affected by the overall iron levels in the organism as a whole, nor by excessive availability of the element. In Hfe mice, which are model animals for human homeostasis, with excessive dietary absorption of iron, excessive iron content in the brain was not observed despite the overall systemic iron overload [57]. The study suggests that iron metabolism in the central nervous system is characterized by considerable autonomy, although it remains, to some extent, regulated by the post-transcriptional mechanisms controlled by IRP1 and IRP2 proteins [58]. At the same time, it has also been demonstrated that correct brain function requires continuous access to appropriate amounts of iron in the early, neonatal phase of ontogenesis [59]. This is probably due to the hypomyelination of nerve fibers in the brain and the medulla oblongata, which is related to both the activity of iron-dependent enzymes of the respiratory chain synthesized by oligodendrocytes and the activity of the enzymes partaking in the synthesis of cholesterol and fatty acids, myelin precursors, whose cofactors contain iron. 

### 4.2. Toxic Heavy Metals 

Under the influence of toxic heavy metals, the concentration of ROS in the organisms increases [60], which may be due to the release of transition metals from their natural locations in cells. The pathophysiological of toxic heavy metals result primarily from intensification of free radicals’ production or weakening of the organism’s defense mechanisms. The oxidative stress induced by toxic metals reduces the efficiency of the antioxidative defense system (reduced activity of antioxidative enzymes and endogenous non-enzymatic antioxidants, as well as reduced concentration of antioxidative vitamins), which results in damage to systems and organs [10,61,62,63]. Toxic metals are not involved in Fenton’s reactions, i.e., they do not directly rigger increased production of free radicals. Indirectly, however, they facilitate the emergence of oxidative stress through secondary contribution to more intensive lipid peroxidation, damage to nucleic acids, changes to gene expression and apoptosis processes, inhibition of the activity of antioxidative proteins by bonding to their sulfhydryl groups, and disorders of calcium homeostasis [10,60,61,64,65]. Studied conducted on rats revealed that short-term exposure to small doses of toxic metals results in the stimulation of antioxidative processes in the brain, but long-term exposure eventually leads to failure of antioxidative mechanisms [66]. 

The primary mechanisms of the prooxidative activity of Pb include (1) a direct influence on the structure and function of cell membranes, particularly in terms of erythrocytes, which leads to their higher susceptibility to oxidative damage; and (2) the Pb-catalyzed autooxidation of hemoglobin, which may initiate peroxidation of unsaturated fatty acids in erythrocyte membranes [67]. It has also been demonstrated that due to Pb’s interference with heme synthesis, the d-aminolaevulinic acid (ALA) accumulating in organs (particularly liver and bone marrow) becomes the source of ROS, which results in oxidative damage [68]. The studies conducted by Sandhir et al. [69] and Sainath et al. [70] revealed that exposing rats to Pb leads to increased lipid peroxidation in the brain and inhibition of antioxidative enzymes’ activity, which is associated with the oxidative inactivation of enzymes and accumulation of peroxides due to either acute or chronic poisoning with toxic substances. Reduced SOD activity may be due to slower synthesis of the enzyme, oxidative changes to the enzymatic protein’s molecules and/or inactivation of the active enzyme center. The substitution of cadmium SOD in place of Zn (in Cu/ZnSOD) or Mn (in MnSOD) reduces the activity of this enzyme [71]. It has been observed that the activity of SOD is inhibited by the enzymatic protein molecules’ reaction with free radical [72]. SOD is closely correlated with CAT which catalyzes the reaction of hydrogen peroxide disproportioning [73]. Lowered CAT activity may be due to the molecules of this enzyme being modified by hydrogen peroxide accumulating inside cells.

The key factor in the toxic activity of Cd stems from the depletion of cellular GSH reserves and total thiol pools (compounds preventing oxidative stress), which inhibits the synthesis of mitochondrial adenosine triphosphate (ATP) and causes insufficiencies in energy production [10]. The activity of Cd is also influenced by the location and concentration of other metals in the organism, primarily Cu, Fe, and Se. The effects of cadmium are non-specific, and the increased lipid peroxidation and functional inhibition of antioxidative enzymes have been observed in numerous *in vivo* and *in vitro* studies [74]. Moreover, human and animal organisms exposed to Cd have been reported to show decreased levels of antioxidative vitamins [61,75]. The participation of Cd in free radical processes is evidenced by the fact that the supply of exogenous antioxidants (e.g. tannic acid, a-tocopherol, ascorbic acid, zinc, selenium) can inhibit the metal’s toxic effects and increase the organism’s overall antioxidative potential [35,76,77]. After short-term exposure of rats to Cd, increased activity of antioxidative enzymes in the animals’ tissues was observed, which may indicate the cells’ attempt to adapt to the conditions of strong oxidative stress [64,66]. 

The toxicity mechanisms of arsenic (As) and its contribution to oxidoreductive processes have yet to be fully explained; however, as suggested by the published studies, the metabolism of arsenic triggers the production of free oxygen radicals, particularly the superoxide radical and hydrogen peroxide, as well as a reduction in the concentration of antioxidative vitamins [61,78]. Arsenic has the capacity for non-specific reactions with the thiol groups in proteins, in particular glutathione and cysteine, which leads to a secondary disturbance of their activity [78]. The depletion of GSH reserves may result in a rapid intensification of free radicals’ production and oxidative damage to biomolecules [79]. A study on the brains of rats exposed perinatally to as (2 or 4 mg kg^−1^ body mass) revealed changes resulting from reduced expression of mRNA and DA-D2 receptor protein [80]. The cited authors also observed the expression of tyrosine hydroxylase and a decrease in the levels of dopamine and its metabolites in the striatum, as well as changes in the frontal cortex and the hippocampus, which led to motor problems and reduced learning and memory capacity. 

Studies indicate that the neurotoxicity of heavy metals may be due to the activity of immunological and proinflammatory factors such as interleukin-6 and bacterial endotoxin, which increase their penetration into the neurons of the central nervous system [81]. 

## 5. The Inhibitory Effects of Exogenous Antioxidants on the Processes of Oxidation 

**Figure 2:**
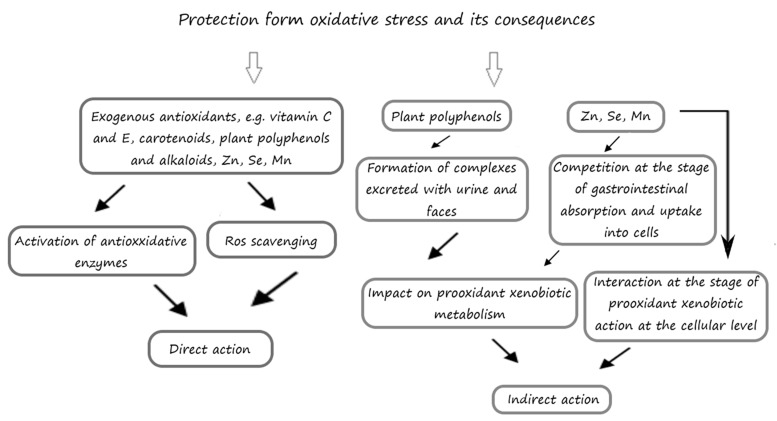
Possible mechanism of exogenous antioxidants action.

Oxidative stress reduces the efficiency of the organism’s antioxidative defense system, whereas the use of exogenous antioxidants reduces the likelihood of oxidative damage (Figure 2). The effects of exogenous antioxidants can be twofold. Firstly, they act synergistically, trapping oxygen and chelating prooxidative metals by catalyzing oxidation reactions [82,83,84]. This activity entails the donation of hydrogen to phenoxy radicals, which restores their antioxidative properties. This group of antioxidants includes substances capable of trapping oxygen such as: ascorbic acid, ascorbyl palmitate, metal chelating compounds, e.g., citric acid, and other secondary antioxidants—amino acids, flavonoids, vitamin A, beta-carotene, selenium, and many others. Secondly, antioxidants may stop radical reactions by donating hydrogen atoms (HAT—hydrogen atom transfer) or electrons (SET—single electron transfer), which transforms the radical into a more stable compound [83,85,86]. The capacity of an antioxidant to donate a hydrogen atom is determined by its bond dissociation energy (BDE). A reaction is possible if he antioxidant’s BDE is lower than that of the reduced radical form. Therefore, the lower the BDE, the stronger the antioxidative potential of a given compound [87]. Mixed reaction mechanisms can also occur between radicals and antioxidants, e.g., involving proton-coupled electron transfer (PCET), sequential proton-loss electron transfer (SPLET), or electron transfer—proton transfer (ET-PT) reactions [88]. The group of compounds whose activity can be classified as the above includes phenols such as gallates, hydroquinones, trihydroxy-butyrophenones, and tocopherols. 

### 5.1. The Influence of Exogenous Antioxidants on the Cerebral Antioxidative Status in Laboratory Animals

In the course of evolution, living organisms developed a range of enzymatic and non-enzymatic defense mechanisms whose aim is to keep ROS at low levels harmless to cells [10]. The most important of such defense mechanisms take advantage of the antioxidative properties of SOD, CAT, GPX, and GST (glutathione transferase). Short-term exposure to oxidants increases the activity of SOD, CAT, PGX, and glutathione reductase, which indicates the activation of defense mechanisms and cellular adaptative response. Under longer-term exposure, cells show a significant decrease in the activity, which is due to the dislodgement from the active MnSOD center of Mn, Cu, and/or Zn ions in the case of Cu/ZnSOD, Fe from the hemic catalase system, or Se ions from glutathione peroxidase [68,89,90]. Studies show that the use of exogenous antioxidants with low molecular mass facilitates the efficiency of the organism’s antioxidative system (Table 1), which reduces the likelihood of oxidative damage being induced in the brain. 

#### 5.1.1. Phenolic Compounds

The antioxidative activity of flavonoids entails: (1) trapping ROS, (2) limiting ROS production by inhibiting the activity of oxidative enzymes and chelating trace elements, and (3) increasing the effectiveness of endogenous antioxidants [121]. The low redox potential of flavonoids, due to proton donation, allows them to reduce strongly oxidized free radicals such as peroxides, alkoxyl, hydroxyl, and peroxidic radicals [122]. 

Numerous studies have been conducted that evidenced the antioxidative influence of phenolic compounds in organisms exposed to substances triggering cerebral oxidative stress (Table 1). In an experiment conducted on rats poisoned with Cd and Pb, the activity of SOD increased only after 12 weeks from the oral application of tannic acid (after 6 weeks, increased activity was observed only for CAT), which suggests that in the case of the brain, long-term and systematic applications of polyphenols is more effective [66]. In turn, in a study by Ashafaq et al. [91], a significant improvement was reported in terms of the antioxidative status in the brains of rats poisoned with Pb, already after two weeks from the application of tannic acid. Tannic acid also antioxidative properties that are equally strong to those of BHA, BHT, and α-tocopherol, and should therefore be considered as a health-beneficial factor naturally found in food [66]. Quercetin is a phenolic compound present in, e.g., tea, whose antioxidative properties have been conclusively demonstrated [123]. The conducted studies confirmed that the use of quercetin in rats poisoned with toxic substances improved cerebral oxidative parameters [94,97]. Moreover, Dong et al. [95] demonstrated that the use of quercetin in rats whose brain damage resulting from subarachnoid hemorrhage triggered increased SOD activity and GDH content and decreased MDA concentration in the brain. Catechins are bioactive ingredients of tea with antioxidative and anti-inflammatory properties [124]. Haque et al. [125] reported that long-term (26 weeks) administration of catechins isolated from green tea (0.5% aqueous solution) prevented β-amyloid-induced cognitive disorders in rats. Moreover, the authors demonstrated that the levels of lipid peroxides in the hippocampus and plasma, and ROS were over 20% lower than in the control group. The antioxidative properties of catechins result from both their radical activity and their ability to chelate the prooxidative Fe^3+^ ad Cu^2+^ ions (which inhibits Fenton’s reaction) [126]. It is known that said ions accumulate in the brains of patients suffering from AD [127]. Similar results were reported by Biasibetti et al. [128] in rats with from streptozotocin-induced dementia. Epigallocatechin gallate (EGCG) found in tea, particularly green tea, is a flavonoid characterized by very string antioxidative properties. A study revealed a very significant increase in the enzymatic and non-enzymatic antioxidative properties as well as a decrease of oxidative stress indicators in the brains of rats poisoned with NaF after a 4-week application of EGCG [92]. Numerous other studies available in the literature confirm the positive effects of green tea in cases of oxidative stress in the brains of laboratory animals poisoned with prooxidative substances (Table 2). The positive influence of green tea extract on the activity of antioxidative enzymes in the brains of rats poisoned with Pb dosed at 100 mg kg^−1^ body mass for 15 days was reported by Khalaf et al. [129]. The authors additionally observed a reduction in DNA damage in the brains of rats receiving a green tea infusion. Additionally, the studies by Meki et al. [130] and Hamed et al. [62] demonstrated increased activity of endogenous antioxidants and reduced levels of oxidative stress indicators in the brains of rats poisoned with lead and simultaneously receiving green tea infusion. Hegazi et al. [103] reported that in the brains of rats poisoned with inhaled prooxidative gasoline, the applied green tea extract proved more efficient in improving the antioxidative status than curcumin. Studies also confirmed the positive effects on oxidative stress (increased SOD, CAT, GSH, GPX) in the brains of rats poisoned with Pb and Cd after the application of not only green but also black, red, and white tea [35]. Simultaneously, in the latter study it was emphasized that the effectiveness of tea increased with longer duration of the experiment (6 vs. 12 weeks), which may suggest that the best effects are obtained through continuous, long-term consumption of those beverages. The cited studies indicate that phenolic compounds contained in tea may reduce oxidative stress in peripheral and cerebral tissues, as well as inhibit behavioral changes related to cognitive deficits. These results suggest that tea, which is the most popular antioxidant rich drink worldwide, should be thoroughly studied in the context of the prophylactics of neurodegenerative diseases. 

Resveratrol, a phytochemical polyphenolic compound naturally occurring in many species of plants, prevents oxidative nerve damage, as demonstrated in studies conducted on rats [119]. In the brains of rats poisoned with AlCl_3_ and NaF, and receiving resveratrol, in improvement in neuron structure was observed, as well as a reduction of the vacuolar spaces surrounding nerve cells and neurofibrillary tangles when compared to rats not receiving resveratrol. Moreover, lowered MDA and increased CAT and SOD content was observed in the brains of rats receiving resveratrol compared to the control (Table 1). Additionally, in the study by Turkmen et al. [120], a positive impact of resveratrol was observed in terms of oxidative stress in rats poisoned with an herbicide containing glyphosate (Table 1). Improvement in the antioxidative status of rats was also observed in the course of studies during which rats received grape seed extract (Table 2). The positive effects were due to the presence of many strongly antioxidative substances in the seeds, including resveratrol and catechins, as well as procyanidin, phenolic acids, tocopherol, and carotenoids [145]. 

Curcumin demonstrates antioxidative and anti-inflammatory activity. It has the ability to quench ROS, influences the concentration of glutathione and activation of antioxidative enzymes. Curcumin shows high affinity for fats and the ability to cross the blood-brain barrier [146]. In the brain, curcumin facilitates the reduction of β-amyloid aggregation [147]. It is characterized by low bioavailability, which means it is not toxic even when consumed in large amounts [148]. Despite its low bioavailability, experimental studies confirm the high antioxidative potential of curcumin and elderly persons as well as those at risk of neurodegenerative diseases are recommended to include it in their diet [149,150]. Studies conducted on laboratory animals confirmed the positive effects of curcumin on cerebral antioxidative status (Table 1). In Swiss mice poisoned with prooxidative lead acetate, it was observed that curcumin has positive effects at the level of neurotransmitters in the hippocampus, where increased levels of serotonin and decreased levels of dopamine were reported, while no such effects were observed in the striatum [151]. Similar results were observed in the brains of rats poisoned with K_2_Cr_2_O_7_ [102] and (CH_3_COO)_2_Pb [101].

Morin, a flavanol found in many plant species, is characterized by high bioactivity as it interacts with nucleic acids, enzymes, and proteins [111]. The conducted studies revealed an increase in both enzymatic and non-enzymatic antioxidative parameters and a decrease in oxidative stress parameters in the brains of rats poisoned with prooxidative substances (Table 1). Similar results were obtained in laboratory animals receiving propolis reach in polyphenols and other antioxidants (Table 1). It was demonstrated that adult rats poisoned with lead and receiving propolis showed an increase in vitamin C and E content in the brain, i.e., an increase in antioxidative potential, as well as a decrease in terms of lipid peroxidation [137]. Due to its antioxidative properties, Propolis shows strong neuroprotective capacity [147]. It was demonstrated that the phenethyl ester of caffeic acid, one of the main ingredients of propolis, increases the release of gastric acid stimulated by the receptor agonist of acetylcholine, probably by inhibiting the activity of AChE [137].

Many types of fruit contain phenolic compounds and are therefore characterized by strong antioxidative properties. Studies have been conducted where rats were poisoned with prooxidative metals received fruit extract or juices (Table 2). It was demonstrated that the use of extracts or juices of pomegranate, strawberries, watermelon, and *Garcinia Indica* fruit, which are popular in Hindu medicine, led to an improvement in cerebral antioxidative potential. The antioxidative properties of fruit result from the presence of large amounts of phenolic compounds, primarily phenolic acids and catechins, epicatechins, flavan-3-ols, kaempferol, quercetin, and luteolin [132,152]. Apart from the same, the mentioned fruit also contain a number of other substances with known antioxidative properties: vitamins C and E, β-carotene, selenium, and anthocyanins [132,153]. Moreover, red-flesh watermelons also contain lycopene, as well as saponins and tannins [143]. A study by Oyenihi et al. [143] was conducted on rats receiving watermelon juice to balance the prooxidative effects of ethanol and revealed an improvement in cerebral antioxidative parameters (↓ MDA, ↑ GSH) as well as normalization of CAT activity. In the cited experiment, the CAT level increased rapidly in the brains of rats exposed to ethanol, which may indicate a cellular attempt to functionally adapt to the conditions of strong oxidative stress. Catalase oxidizes ethanol in the presence of a system producing hydrogen peroxide with the formation of acetaldehyde [154]. Consequently, the increased activity of CAT after acute exposure to ethanol signifies intensive oxidation. 

Carvacrol, a monoterpene phenol produced by various species of herbs, shows strong antioxidative properties, as demonstrated in studies conducted on rats kept under conditions of chronic stress [117]. The brains of rats receiving H_2_O_2_, after the administration of *Thymus algeriensis* extract, showed an increase in the enzymatic and non-enzymatic parameters of oxidative stress and a decrease in LPO and NO (Table 2), which may be due to the presence of carvacrol as well as other phenolic compounds [155]. The presence of phenolic compounds (apigenin, luteolin, kaempferol, myricetin, quercetin, caffeic acid) as well as antioxidative vitamins in the extract from parsley leaves caused a decrease in MDA observed in the brains of rats receiving d-galactose which shows proinflammatory properties [144].

#### 5.1.2. Alkaloids

Studies have confirmed the protective properties of alkaloids relative to oxidative damage induced by free radicals [156,157]. Caffeine present in coffee blocks the receptors of adenosine A1 and A2 and contributes to increased activity of the central nervous system [158]. The blocking of adenosine A1 receptors causes an increase in the release of neurotransmitters such as acetylcholine, noradrenaline, and dopamine. The inactivation of adenosine A2 receptors leads to increased dopamine activity and its stronger bonding to the D2 receptor, it also protects dopaminergic receptors against toxic factors [158,159]. The influence of caffeine on dopamine activity may be significant in AD and PD [19,160]. A study conducted by Khan et al. [118] demonstrated that administration of caffeine (30 mg kg^−1^ per day) mitigated neurodegenerative lesions in the brains of mice poisoned with Cd as well as improved their cognitive functions and memory. Additionally, the cited authors studied the cerebral antioxidative status and degree of neuron apoptosis *in vivo* and *in vitro* on BV-2 cell lines. The antioxidative influence of alkaloids on the brains of rats has been confirmed in multiple studies. Czapski et al. [161] observed that alkaloids isolated from *Huperzia selago* and *Diphasiastrum complanatum* provided significant protection against lipid peroxidation and protein oxidation in the rat brain, reducing the damage caused by iron by approximately 20%. Moreover, the same authors reported that alkaloid extracts concentrated at 25 μg mL^−1^ demonstrated strong antioxidative properties with the potential to trapradicals exceeding 59%. They also observed a reduction in dityrosine production. 

The study by Almeida et al. [162] performed with the use of isolated cells of a C6 glioma exposed to H_2_O_2_ and/or morphine for 24 hours, revealed that morphine, administered in standard analgesic doses, may contribute to the minimization of oxidative stress in glia cells. Admittedly, in the cited studies, morphine did not prevent the reduced survivability of cells exposed to H_2_O_2_ but did partially prevent lipid peroxidation and did not cause an increase in the level of sulfhydryl groups in the studied cells due to the H_2_O_2_ exposure. Morphine is capable of activating the family of type µ opioid receptors (for morphine), λ (for ketocyclazocine), and κ (for diamorphine) [163]. Endogenous opioids are present in a number of structures of the central nervous system and spinal medulla. The classic analgesic effects of morphine are associated primarily with the activation of µ-receptor, which leads, among other effects, to inhibition of the calcium flow in presynaptic neurons and increased potassium conduction in postsynaptic neurons. Opioid receptors are located in, e.g., glia cells, particularly microglia and astrocytes, where they can participate in, e.g., pathological neurotropism [162]. In embryos of zebrafish *(Danio rerio)*, morphine dosed at 1, 10, or 100 nM increased neuron proliferation, the populations of some neurons, and prevented irregularities in glutamate metabolism in motor and Pax-6 neurons [164]. Glutamate metabolism disorders are associated with cognitive impairment [165]. A study conducted by Cui et al. [166] revealed that morphine, as well as endomorphine-1 and endomorphine-2, may provide protection against the intracellular toxicity of amyloid in primary human and animal neuron cultures as well as in rat brains *in vivo*. Moreover, the authors observed that morphine improved the spatial memory of rats and mice in the Morris water maze test, which, according to the cited authors, was due to the morphine-provoked induction of estradiol release in hippocampal neurons. 

In an *in vivo* study conducted on apoplectic rats receiving leonurine, an alkaloid present in *Herba Leonuri*, the authors observed an improvement in terms of the neurological deficit, increased activity of antioxidative enzymes, as well as reduced MDA levels in the brains of the animals [116]. Moreover, leonurine inhibited mitochondrial production of ROS and the biosynthesis of adenosine triphosphate. 

#### 5.1.3. Vitamins and Provitamins

Vitamin C is produced in the process of oxygen metabolism and quickly reacts with singlet oxygen, ozone, and H_2_O_2_ in the process of ascorbic peroxidase, which allows it to neutralize their toxic effects. In plants, vitamin C also contributes to the regeneration of other strong antioxidants: carotenoids and vitamin E [167]. Vitamin C is capable of crossing the blood-brain barrier and trapping free radicals both intra- and extracellularly. It is considered to be the most important water-soluble antioxidant present in extracellular fluids due to its ability to neutralize RTF in the aqueous phase, before the onset of lipid peroxidation [168]. El-Sokkary and Awadalla [104] reported positive effects of using Vitamin C dosed at 100 mg kg^−1^ body mass on the activity of SOD and GSH in the brains of rats poisoned with Cd dosed at 5 mg kg^−1^ body mass over 40 days (Table 1). The ability of vitamin C to reduce the neurotoxicity of nutmeg in the brains of rats was demonstrated by Salman et al. [169]. In a study by Renugadevi et al. [77] rats received Cd for 284 days (5 mg kg^−1^ body mass daily) as well as vitamins C and E dosed at 50 mg kg^−1^ body mass daily. Among other observations, the authors reported a positive impact of the analyzed vitamins on the activity of endogenous enzymatic and non-enzymatic cerebral antioxidants (SOD, CAT, GPX, GSH) as well as increased activity of adenosine triphosphatases responsible for maintaining cell homeostasis and the process of neurotransmission. A study by Dortaj et al. [170] revealed that acrylamide-induced neurological damage to the cerebellum of rat fetuses could be minimized due to the effects of vitamin C. 

Vitamin E is the main fat-soluble and the most effective chain breaking antioxidant in cell membranes, where it protects fatty acids against lipid peroxidation [171]. Studies conducted on rats poisoned with cadmium [105] or deltamethrin [106] demonstrated an improvement in cerebral oxidoreductive status parameters after administration of the vitamin dosed at 100 or 200 mg kg^−1^ body mass for 1 month (Table 1). In experiments where rats were poisoned with various toxins received selenium as well as from vitamin E, the effects were also present. The studies reported improvement of enzymatic and non-enzymatic parameters serving as indicators of oxidative stress, including increased levels of vitamin C [107] and vitamin E [76]. Supplementation using a combination of vitamin E and selenium decreased TBARS and improved the activity of antioxidative enzymes as well as the GSH level in the brains of rats poisoned with glucocorticoid [108]. A study conducted on rats receiving a high-fat diet for 9 months revealed impairment of spatial memory and increased mortality of hippocampal cells [172]. However, when such diet was enriched with the addition of vitamins C and E, a protective effect of the latter on nerve cells was observed [172].

The antioxidative activity of carotenoids stems from their ability to trap peroxide radicals. The number of conjugated double bonds in these molecules corresponds to their effectiveness in trapping ROS [167]. Astaxanthin (3,30-dihydroxy-β, β-carotene-4,40-dione) is a red pigment belonging to the sub-class of xanthophyllic carotenoids; it is found mainly in microalgae, mushrooms, and complex plants, as well as in salmon, trout, and crustacean cells [114]. Astaxanthin shows strong antioxidative properties and can neutralize singlet oxygen and free radicals, thus, preventing lipid peroxidation [173]. Its properties are due to the presence of numerous conjugated multiple bonds. It has been demonstrated that the most important effects of astaxanthin’s activity include the prevention of neuron damage and inhibition of cell membrane peroxidation [173]. Astaxanthin is composed of a long, nonpolar, conjugated bond connecting polar ion rings located on both ends of the chain. Due to the extended π conjugation, astaxanthin reacts towards many free radical reductions, and the presence of polar ion rings containing hydroxylic and carbonyl groups provides it with higher antioxidative capacity compared to other carotenoids [174]. It has been demonstrated that treading rats with astaxanthin (25 mg kg^−1^ body weight) five times a week for four weeks, with simultaneous administration of high doses of doxorubicin (8 mg kg^−1^ body weight in total for 4 weeks), an anti-cancer agent with proven neurotoxic properties, resulted in improvement of cerebral oxidative stress parameters (Table 1), while behavioral tests demonstrated that the treatment significantly improved memory, restored the histopathological architecture of the hippocampus, limited oxidative and inflammatory damage, and reduced the increase in acetylcholinesterase activity [115]. An increase in GSH and SOD, and a decrease in MDA levels in the brains of rats poisoned with Cd was reported by Akkoyun et al. [114]. In a study by Wang et al. [175], a positive influence of astaxanthin was observed rats whose serum contained elevated levels of homocysteine, which increases the risk of neurodegenerative diseases. The authors reported a reduction in terms of mitochondrial dysfunction in the hippocampus due to, e.g., inhibition of cell apoptosis in primary neurons and inhibition of the release of intracellular RTF and superoxide anions. The antioxidative properties of lycopene, another carotenoid, have also been evidenced in numerous studies [176,177]. In the most recent study with the use of animal models, it was demonstrated that lycopene acts neuroprotectively by mitigating oxidative stress, inhibiting the production of inflammatory cytokines, preventing the accumulation of amyloid plaques, inhibiting neuron apoptosis, and restoring mitochondrial function [178]. Moreover, it was observed that lycopene mitigated cognitive deficits by affecting the inflammatory state along the intestines–liver–brain axis and improving the metabolism of glycolipids [99,179]. In a study conducted by Wang et al. [99] on mice receiving proinflammatory and antioxidative liposaccharide, it was observed that apart from improving cerebral antioxidative status, lycopene can also mitigate inflammations occurring in the nervous system, amylogenesis and cognitive impairment, most likely by mediating the signal pathways for MAPK (mitogen-activated protein kinases), nuclear factor κB, and Nrf2 (nuclear factor-erythroid 2-related factor 2). Behavioral tests, including Y maze test, locomotor activity, and Morris water maze test, revealed that long-term supplementation with lycopene (50 mg kg^−1^ body mass daily) mitigated the cognitive disorders induced with d-galactose in male CD-1 mice [100]. The use of lycopene prevented histopathological damage and restored the levels of the neurotrophic factor originating from the brain in the mice’s hippocampus. In mice receiving lycopene, a significant increase in the activity of antioxidative enzymes was observed, as well as a decrease in the inflammatory cytokines’ level in the serum as compared to rats receiving d-galactose. The use of lycopene-rich tomato extract in rats poisoned with prooxidative Cd resulted in an increase in SOD, CAT, and GSX activity, with simultaneous decrease in MDA activity in the animals’ brains [142].

## 6. Antioxidative Therapies in Neurodegenerative Diseases—Clinical Studies

Clinical studies pertaining to antioxidative therapies with potential application to neurodegenerative diseases have been very intensive in the recent years. In 2018 alone, in the USA and many European and Asian countries, over 10 clinical studies were conducted that pertained solely to the impact of catechins on the development of AD [126]. Simultaneously, other antioxidants and other neurodegenerative diseases are also being analyzed.

In a study conducted in Japan by Kuriyama et al. [180] in a group of 1003 persons aged > 70 years, it was demonstrated that the incidence of cognitive disorders was lower in persons consuming more green tea. Additionally, a study conducted in China among subjects aged over 65 years revealed a negative correlation between consumption of green tea and incidence of cognitive disorders [181]. Similar results were reported in Norway [182] and Singapore [183]. However, Huang et al. [184] and Arab et al. [185] showed that this tendency only applies to men. A 7-year study conducted in China in a group of 7000 subjects aged at least 80 years also revealed lesser cognitive disorders in persons regularly consuming green tea [186]. In a study by Noguchi-Shinohara et al. [187] conducted in a group of 70-year-old or older subjects a similar correlation was observe, although the authors simultaneously concluded that consumption of black tea or coffee does not decrease the risk of dementia. In turn, Ng et al. [188] observed that drinking black and oolong tea can improve cognitive functions in elderly persons. A study conducted in a group of 75 AD patients and 75 control subjects revealed that each additional glass of tea consumed daily reduces the risk of PD approximately 0.8 times [189]. A study conducted in Canada in a group of 4600 adults revealed a 31% reduction of the risk of AD in persons who drank coffee on a daily basis [190]. However, results reported in this type of studies are somewhat ambiguous: some suggest a correlation between drinking coffee and increased risk of AD, while others report a reverse correlation between consumption of coffee and the risk of the disease. Nonetheless, most studies seem to suggest that moderate consumption of coffee during the middle-age may reduce the risk of dementia and AD later in life [191].

Kataoka et al. [192] conducted a study during which patients received powdered tea capsules for a period of 12 months. The experiment revealed a positive influence of green tea on cognitive functions. Ide et al. [193] conducted a study among 12 persons aged over 65 years and diagnosed with cognitive disorders. The subjects received powdered green tea dosed at 2000 mg daily. In the cited study, significant improvement was observed as early as three months into the treatment. The same team conducted a study involving 17 persons receiving 2000 mg of green tea powder every day for 12 months. The researchers observed a decrease in the level of one of key markers of oxidative stress in AD patients—MDA-LDL (a low-density aldehyde-modified lipoprotein) [124]. 

Certainly, the effectiveness of such activity is influenced by the duration of treatment, as well as the dosage and form in which antioxidants are administered. In a study by Park et al. [194], 91 persons received diet supplements containing 1440 mg of green tea extract for 16 weeks. The study did not reveal any differences between subjects receiving the supplement or the placebo in terms of memory or selective attention; possibly, the results would be more apparent in a longer supplementation period. Chen et al. [195] concluded that the effects of green tea can be observed if it is consumed at least three times a week for a minimum of 6 months. In turn, Fischer et al. [196] did not report enhanced cognitive functions in the German population (418 AD patients aged over 75 years) consuming greater amounts of green tea, which is explained by the authors by the irregular consumption, short time of consumption (this is a novel non-traditional foodstuff), and genetic background.

Gao et al. [197] analyzed the correlation between regular flavonoid-rich diet and the risk of PD in over 49 thousand men and over 80 thousand women. The cited authors concluded that diets rich in EC dimers and proanthocyanidins correlated with a lower risk of PD. In the authors’ opinion, this is due to the fact that when EC are supplied to the organism on a regular basis, they can stimulate the phosphorylation of the CREB transcription factor (cAMP response element-binding protein), which serves as the regulator of neuron vitality and synaptic plasticity, with the cAMP factor (3’,5’-cyclic adenosine monophosphate), as well as inhibit the activity of NADPH oxidase. Furthermore, the authors posited that proanthocyanidins may increase the concentration of dopamine in the brain and inhibit the activity of monoamine oxidase A. 

Single studies with human participants have demonstrated a reduction of neurodegenerative symptoms after application of ketogenic diet [198,199]. The presence of ketone bodies has potent mitochondrial effects modulating the expression of enzymes involved in mitochondrial metabolism and increasing mitochondrial biogenesis in key brain regions such as the hippocampus. Under the influence of ketone bodies, the concentration of ROS in the organisms decreases, while the biosynthesis of GSH and SOD2 increases [198,200].

## 7. Conclusions

The correlation between oxidative stress and neurodegenerative diseases has been suggested in a growing number of studies, although it has yet to be conclusively determined whether oxidative stress is the cause or symptom of damage to the central nervous system. However, based on the analysis of available literature, it can be posited that in order to delay or mitigate the progression of neuronal degeneration, one should take steps to ensure proper antioxidative status of the organism. Future research will most likely pertain to potential combinations of pharmacological and dietary provision of antioxidants to patients at risk of developing neurodegenerative diseases. Chen et al. [123] analyzed a combined therapy with the use of memantine polyphenols and tea. In the cited study, the combined therapy proved more effective in defending cells against cytotoxic damage and motor impairment than any of the ingredients used independently. In turn, Zhang et al. [201] employed a combination of catechins and acetylcholinesterase inhibitors. Researchers increasingly lean towards the conclusion that studies should now primarily focus on combining medical and dietary methods to develop the most efficient approach to the treatment and prevention of neurodegenerative diseases. Individual optimization of the intake of exogenous antioxidants and dietary supplements for each patient, depending on the status and function of the organism, is the key strategy.

## Figures and Tables

**Table 1:** Antioxidative influence of food ingredients on the brain.

	Protective Effect	Design	Animals	Target Sites	References
Tannic acid	↑ SOD after 12 weeks; ↑ CAT both after 6 and 12 weeks	7 mg Cd (as cadmium chloride) and 50 mg Pb (as lead acetate) per kg of feed for 6 or 12 weeks; tannic acid with drink (0, 0.5, 1, 1.5, 2 or 2.5% solutions) for 6 or 12 weeks	Male Wistar rats	Total brain	[66]
	↑ SOD after 12 weeks; ↑ CAT both after 6 and 12 weeks	aqueous solutions of [Cd (7 or 14 mg L^−1^ distiller water) or Pb (50 or 100 mg L^−1^ distilled water)] or 2 % tannic acid solution, alternatively every 7 days, for 6 or 12 weeks			
Tannic acid	↓ LPO; ↑ GSH; ↑ GST; ↑ GPX; ↑ SOD; ↑ CAT	50 mg kg^−1^ bw lead acetate intraperitoneally three times a week for two weeks; 50 mg kg^−1^ bw tanic acid orally three times a week for two weeks	Male Wistar rats	Total brain	[91]
Epigallocatechin gallate (EGCG)	↑ CAT; ↑ SOD; ↑ GPX; ↑ GSH; ↑ GST; ↑ GR; ↑ G6PD; ↑ TSH; ↓ ROS; ↓ TBARS; ↓ NO; ↓ PC; ↑ vitamin C	25 mg kg^−1^ bw fluoride (as NaF)) per day by intragastric administration for 4 weeks; 40 mg kg^−1^ bw EGCG administrated 30 min before administration of NaF per day by intragastric administration for 4 weeks	Male Wistar rats	Hippocampus	[92]
Quercetin	↓ LPO; ↑ CAT; ↑ SOD; ↑ GPX	Mice with traumatic brain injuries; 20 mg kg^−1^ bw quercetin through intraperitoneal injection for 7 days	Mice	Total brain	[93]
Quercetin	↓ MDA; ↑ CAT; ↑ SOD; ↑ GPX	1 mg Cd (as cadmium chloride) kg^−1^ bw per day by injection for 30 days; 15 mg quercetin kg^−1^ bw orally for 30 days	Male Sprague-Dawley rats	Total brain	[94]
Quercetin	↓ MDA; ↑ SOD; ↑ GSH	Rats with brain damage after subarachnoid hemorrhage; 10 or 50 mg kg^−1^ bw quercetin administered intraperitoneally at 30 min, 12 h, and 24 h after the subarachnoid hemorrhage insult	Male Sprague-Dawley rats	Cerebral cortex	[95]
Quercetin	↑ GSH; ↓ NO	80 mg kg^−1^ ifosfamide intraperitoneally for 5 consecutive days; 50 mg kg^−1^ bw quercetin orally for 6 consecutive days	Adult female rats	Cortex, cerebellum, striatum, pons, thalamus, hypothalamus	[96]
Quercetin	↑ CAT; ↓ MDA; ↑ GPX; ↑ total thiol;	10 mg kg^−1^ chlorpyrifos orally once a day by gavage for 1 month, 30 min after administration of quercetin; 20 mg kg^−1^ quercetin orally once a day by gavage for 1 month	Male Sprague-Dawley rats	Total brain	[97]
Lycopene	↓ MDA; ↑ TAC; ↓neuronal cell death	Diabetic rats; 4 mg kg^−1^ lycopene orally for 8 weeks	Male Wistar rats	Hippocampus	[98]
Lycopene	↑ GSH; ↑ CAT; ↑ SOD	0.25 mg kg^−1^ per day lipopolysaccharide by injection for 9 days; 0.03% lycopene mixed with standard diet for 5 weeks	Male C57BL/6J mice	Total brain	[99]
Lycopene	↓ MDA; ↑ GSH; ↑ SOD; ↑ GPX	150 mg kg^−1^ per day D-galactose by intraperitoneally injection for 8 weeks; 50 mg kg^−1^ bw lycopene per day mixed with standard diet for 8 weeks	CD-1 male mice	Hippocampus	[100]
Curcumin	↑ GSH; ↓ TBARS	25 mg kg^−1^ lead acetate orally for 2 weeks; alone and after 1 h treated orally either with curcumin (15 mg kg^−1^) or nanocurcumin (15 mg kg^−1^) for 2 weeks	Swiss albino mice	Total brain	[101]
Curcumin	↑ GSH; ↑ SOD; ↑ GPX; ↑ GR; ↑ GST	15 mg kg^−1^ bw potassium dichromate by a single intraperitoneal injection on 10 days; 400 mg kg^−1^ bw curcumin orally for 10 days	Male Wistar rats	Total brain	[102]
Curcumin	↑ CAT; ↑ SOD; ↑ total thiol; ↓ TBARS; ↓ AOPP; ↓ PC	Gasoline inhalation—2 hours daily; 3% powdered curcumin roots in feed	Male mice CD1 strain	Total brain	[103]
Vitamin C	↓ MDA; ↑ SOD; ↑ GSH	5 mg kg^−1^ bw cadmium chloride injected subcutaneously every day for 49 days; 100 mg kg^−1^ vitamin C injected subcutaneously every day for 49 days 30 min. before Cd injection	Male Sprague-Dawley rats	Total brain	[104]
Vitamin C, vitamin E	↓ LPO; ↑ AChE; ↑ SOD; ↑ CAT; ↑ GPX; ↑ GSH; ↑ ATPases	5 mg kg^−1^ bw per day cadmium chloride orally for 284 days; 50 mg kg^−1^ bw per day vitamin C and vitamin E orally for 248 days	Rats	Total brain	[77]
Vitamin E	↓ MDA; ↓ NO; ↑ TAC	0.6 mg kg^−1^ bw deltamethrin taken once daily via oral gavage for 30 days; 200 mg kg^−1^ bw vitamin E taken once daily via oral gavage for 30 days	Male albino rats	Total brain	[105]
Vitamin E	↑ CAT; ↑ GPX	Waterpipe tobacco smoke exposure for one-hour session per day for five days per week for 1 month; 100 mg kg^−1^ vitamin E once a day by oral gavage for 1 month	Adult Wistar rats	Hippocapus	[106]
Vitamin E, selenium	↓ AOPP; ↑ vitamin C; improved the diminished activities of antioxidative enzymes and the levels of GSH	0.2 g L^−1^ drinking water dimethoate; 100 mg kg^−1^ diet vitamin E; 0.5 mg kg^−1^ diet selenium	Adult Wistar rats	Cerebral cortex tissue	[107]
Vitamin E, selenium	↓ TBARS; improved the diminished activities of antioxidative enzymes and the levels of GSH	100 mg kg^−1^ bw prednisolone injected intramuscularly for 3 consecutive days; 20 mg DL-α-tocopheryl acetate and 0.3 mg sodium selenite for 30 days by oral route	Male Wistar rats	Total brain	[108]
Vitamin E, selenium	↑ SOD; ↓ TBARS; ↑ GSH; ↑ GST; ↑ GR; ↑ vitamin E	20 mg L^−1^ AgNO_3_ in drinking water; 400 mg kg^−1^ diet vitamin E and mg L^−1^ selenium in drinking water	Male Wistar rats	Total brain	[76]
Melatonin	↑ AChE; ↓ TBARS; ↑ GSH; ↑ vitamin C; ↑ vitamin E; ↑ TSH	5 mg kg^−1^ bw cadmium chloride orally for 4 weeks; melatonin (10 mg kg bw) in ethanol subcutaneously for 4 weeks; the injection of melatonin was 30 min before Cd administration	Male Wistar rats	Total brain	[75]
Melatonin	↓elato ↓elat ↑elato ↑ CAT; ↑ SOD; ↑ GPX; ↑ GR	34 mg kg^−1^ bw aluminum chloride orally every day for 7 days; melatonin (10 mg kg^−1^ bw) administered intraperitoneally every day for 7 days; the injection of melatonin was 60 min before aluminum administration	Male Wistar rats	Total brain	[109]
Morin	↓ MDA; ↑ AChE; ↑ MAO; ↑ CAT; ↑ SOD; ↑ GPX; ↑ GST; ↑ GSH; ↑ vitamin C; ↑ vitamin E	3 mg kg^−1^ bw cadmium chloride injected every day for 21 days; morin alone (40 mg kg^−1^ bw) 1 h before cadmium chloride injection for 21 days	Male Sprague-Dawley rats	Total brain	[110]
Morin	↑ SOD; ↑ CAT; ↓ TBARS; ↑ GSH; ↑ GPX;	100 mg kg^−1^ ammonium chloride by intraperitoneal injections thrice in a week for 8 weeks; 30 mg kg^−1^ morin orally by intragastric tube for 8 weeks	Male Wistar rats	Total brain	[111]
Taurine	↓ MDA; ↑ CAT; ↑ SOD	Diabetes rats; 40 mg kg^−1^ bw streptozotocin by a single intraperitoneal injection; single dose 40 mg kg^−1^ bw, and after 3 days they were injected with taurine at a dose 50 mg kg^−1^ bw for 60 days	Male Wistar rats	Total brain	[112]
Taurine	↓ ROS; ↓ MDA; ↑ GSH; ↑ CAT; ↑ SOD; ↑ GPX; ↑ AChE	Traumatic model cells; cells were treated with 100, 200, or 300 mg l^−1^ of taurine for 72 h	Male Wistar neonatal rats	Cortical tissues	[113]
Astaxanthin	↓ MDA; ↑ GSH; ↑ SOD	40 mg L^−1^ per day cadmium chloride orally for 30 days; 20 mg kg^−1^ per day Astaxanthin by gastric gauge for 30 days	Male Wistar rats	Total brain	[114]
Astaxanthin	↑ GSH; ↑ CAT; ↓ MDA;	2 mg kg^−1^ bw per week doxorubicin injected intraperitoneally (one injection per week) for 4 weeks; 25 mg kg^−1^ per day astaxanthin (5 days per week) orally for 4 weeks	Male albino rats	Hippocampus	[115]
Leonurine	↓ MDA; ↑ GPX; ↑ SOD	Stroke rats group treated with 15, 30 or 60 mg kg^−1^ per day of leonurine orally once daily for 1 week	Male Sprague-Dawley rats	Total brain	[116]
Carvacrol	↓ MDA; ↑ GSH; ↑ CAT; ↑ SOD; ↑ GPX; ↑ GR	Chronic restraint stress was performed using a rodent restrainer made of plexiglas that closely fit to the rats’ body (6 h per day for 21 consecutive days); 20, 30, or 40 mg kg ^−1^ carvacrol for 21 days	Wistar rats	Total brain	[117]
Caffeine	↓ LPO; ↓ ROS	30 mg kg^−1^ caffeine per day for 2 weeks; 5 mg kg^−1^ cadmium chloride per day for 2 weeks	Male C57BL/6N mice	Hippocampus, cortex	[118]
Resveratrol	↓ MDA; ↑ CAT; ↑ SOD;	100 mg kg^−1^ bw aluminum chloride and 10 mg kg^−1^ bw sodium fluoride orally with orogastric tube for 8 weeks; 30 mg kg^−1^ bw resveratrol orally with orogastric tube for 8 weeks	Sprague Dawley rats	Total brain	[119]
Resveratrol	↓ MDA; ↑ GSH	375 mg kg^−1^ glyphosate-based herbicide in distilled water with a gastric gavage once a day for 56 days; 20 mg kg^−1^ resveratrol in distilled water with a gastric gavage once a day for 56 days	Male Wistar rats	Total brain	[120]

↑—increased concentration or activity compared to oxidant-exposed group; ↓—decreased or inhibited concentration or activity compared to oxidant-exposed group; SOD—superoxide dismutase; CAT—catalase; GPX—glutathione peroxidase; GSH—glutathione; GST—glutathione S-transferase; GR—glutathione reductase; TBARS—thiobarbituric acid reactive substances; ROS—reactive oxygen species; NO—nitrite/nitrate; LPO—lipid peroxidation; TAC—total antioxidant capacity; MDA—malondialdehyde; AChE—acetylcholinesterase; ATPases—adenylpyrophosphatase; MAO—monoaminoxidase activity; AOPP—advanced oxidation protein products; TSH—total sulphydryl groups; PC—protein carbonyls; G6PD—glucose-6-phosphate dehydrogenase; bw—body weight.

**Table 2:** Antioxidative influence of food on the brain.

	Protective effect	Design	Animals	Target sites	References
Green, black, red and white tea infusion	↑ SOD; ↑ CAT; ↑ GSH; ↑ GPX	7 mg Cd (as cadmium chloride) and 50 mg Pb (as lead acetate) per kg of feed for 6 and 12 weeks; infusions of teas as a sole source of drink for 6 and 12 weeks	Male Wistar rats	Total brain	[35]
Green tea infusion	↑ SOD; ↑ GST; ↑ GSH; ↓ NO; ↓ LPO	0.4 % aqueous solution of lead acetate orally for 6 weeks; green tea in drinking water (15 g L^−1^) orally for 6 weeks	Male rats	Total brain	[130]
Green tea infusion	↑ TAC; ↑ RGSH; ↑ SOD; ↓ DNA fragmentation	100 mg of lead acetate/kg bw by gastric tube for 1 month; green tea in drinking water (5 g L^−1^) orally for 1 month	Albino male rats	Total rain	[129]
Green tea infusion	↑ GST; ↑ RGSH; ↑ SOD; ↑ TAC; ↓ LPO	0.4 % aqueous solution of lead acetate orally for 6 weeks; green tea in distilled water (15 g L^−1^) orally for 6 weeks	Rats	Total brain	[62]
Green tea extract	↑ CAT; ↑ SOD; ↑ GPX; ↑ GST; ↑ total thiol; ↓ TBARS; ↓ AOPP; ↓ PC	Gasoline inhalation—2 hours daily; 1.5 % green tea extract rally as a sole source of water	Male mice CD1 strain	Total brain	[103]
Pomegranate peel	↓ TBARS; ↓ NO; ↑ SOD; ↑ CAT; ↑ GPX; ↑ GR	34 mg kg^−1^ bw aluminum chloride orally every day for 7 days; pomegranate peel methanolic extract (200 mg kg^−1^ bw) orally every day for 7 days given before aluminum chloride	Female Wistar rats	Total brain	[131]
Strawberry methanolic extract	↓ LPO; ↓ NO; ↑ GSH; ↑ SOD; ↑ CAT; ↑ GPX; ↑ GR	6.5 mg kg^−1^ bw per day cadmium chloride injected intraperitoneally for 5 days; 250 mg kg^−1^ bw per day strawberry methanolic extract orally administered 1hr before cadmium chloride injection for 5 days	Male Wistar rats	Total brain	[132]
Hydrophobic fractions of *Thymus algeriensis*	↑ SOD; ↑ CAT; ↑ GPX; ↑ GSH; ↑ GST; ↓ LPO	0.1 or 1 1 mmol L^−1^ hydrogen peroxide orally for 15 days; 180 mg hydrophobic fractions of *Thymus algeriensis* per kg^-^bw per day dissolved in normal saline orally for 15 days	Male Sprague-Dawley rats	Total brain	[133]
Grape seed extract and pyridoxine	↑ SOD; ↑ CAT; ↑ GPX; ↑ GSH; ↓ MDA	50 mg kg^−1^ bw per day triton injected intraperitoneally for 4 weeks; 300 mg kg^−1^ bw per day grape seed extract orally for 4 weeks; 12 mg kg^−1^ bw per day pyridoxine orally for 4 weeks	Male Sprague-Dawley rats	Total brain	[134]
Grape seed extract	↑ AChE; ↓ MDA; ↑ SOD; ↑ CAT; ↑ GSH	7.5 mg kg^−1^ chlorpyrifos 80% emulsion concentrate in distilled water for 21 days; 100 mg kg^−1^ grape seed extract orally through oral cannula for 28 days; the time interval between chlorpyrifos and grape seed extract administration was 2 h; all animals were sacrificed on day 45	Male Wistar rats	Total brain	[135]
Ashwagandha extract	↑ GSH; ↓ MDA; ↓ NO;	100 mg^−1^ kg AlCl_3_ orally for 30 days; 200 mg^−1^ kg ashwagandha extract orally for 30 days; the time interval between ashwagandha and AlCl_3_ administration was 60 min	Male Wistar rats	Cortex, hippocampus and striatum	[136]
Propolis	↓ MDA; ↓ PC; ↑ vitamin C; ↑ vitamin E; ↑ cytochrome C oxidase	1 mg^−1^ kg bw lead acetate for 4 weeks; 50 mg kg^−1^ bw propolis orally for 4 weeks	Swiss albino rats	Total brain	[137]
Propolis	↓ MDA; ↑ CAT	0.0082 ppm cypermethrin in fresh water for 15 days; 10 ppm propolis in fresh water for 15 days	Rainbo trout Oncorhynchus mykiss	Total brain	[138]
*Mucuna* seeds extract	↑ SOD; ↑ CAT; ↑ GSH; ↓ MDA; ↑ AChE	2.75 mg L^−1^ sodium dodecyl sulphate in fresh water for 15 or 30 days; 15.5 mg kg^−1^ bw *Mucuna* extract injected intraperitoneally for 7 consecutive days	Catfish Heteropneustes fossilisand	Total brain	[139]
Garcinia Indica fruit extract	↓ MDA; ↓ LPO; ↑ SOD; ↑ CAT; ↑ GSH	75 mg kg^−1^ bw cyclophosphamide injected intraperitoneally 24 h before the termination of the experiment; 250 or 500 mg kg^−1^ bw *Garcinia Indica* fruit extract orally for 14 days	Male Wistar rats	Total brain	[140]
Chlorella vulgaris	↑ SOD; ↑ GPX; ↑ GR; ↑ GSH; ↓ LPO	200 mg l^−1^ lead acetate in drinking water for 4 weeks; 20, 50 or 100 g *Chlorella vulgaris* per 1 kg of food for 4 weeks	Male Sprague-Dawley rats	Total brain	[141]
Tomato extract, garlic extract	↓ MDA; ↑ SOD; ↑ CAT; ↑ GSH	6 mg kg^-2^ bw Cd orally for 15 days; 100 mg kg^−1^ bw garlic extract orally for 15 days; 50 mg kg^−1^ bw tomato extract orally for 15 days	Swiss albino mice	Total brain	[142]
Watermelon juice	↓ MDA; ↑ GSH; ↓ CAT	12 mL kg^−1^ ethanol - a single dose orally; 4 ml kg^−1^ watermelon juice orally for15 days before administration of ethanol	Wistar rats	Total brain	[143]
Parsley leaves ethanolic extract	↓ MDA	0.5 mL per day D-galactose injected for 20 days; 40 mg kg^−1^ bw ethanolic extract of parsley leaves injected for 20 days	Albino male mice	Cerebral cortex, hippocampus, cerebellum, corpora quadrigemina	[144]

↑—increased concentration or activity compared to oxidant-exposed group; ↓—decreased or inhibited concentration or activity compared to oxidant-exposed group; SOD—superoxide dismutase; CAT—catalase; TAC—total antioxidant capacity; RGSH—reduced glutathione; GPX—glutathione peroxidase; GSH—glutathione; GST—glutathione S-transferase; GR—glutathione reductase; TBARS—thiobarbituric acid reactive substances; NO—nitrite/nitrate; LPO—lipid peroxidation; TAC—total antioxidant capacity; MDA—malondialdehyde; AChE—acetylcholinesterase; AOPP—advanced oxidation protein products; PC—protein carbonyls; bw—body weight.
